# Recommendations for collection and integration of community-based testing and linkage to care data into national surveillance, monitoring and evaluation systems for HIV, viral hepatitis and sexually transmitted infections: results from the INTEGRATE Joint Action

**DOI:** 10.1186/s12879-021-06499-5

**Published:** 2021-09-13

**Authors:** Laura Fernàndez-López, Irena Klavs, Anna Conway, Tanja Kustec, Mojca Serdt, Sladjana Baros, Danica Valkovičová Staneková, Liis Lemsalu, Iwona Wawer, Piotr Wysocki, Jordi Casabona

**Affiliations:** 1 Health Department, Generalitat de Catalunya, Centre of Epidemiological Studies of HIV/AIDS and STI of Catalonia (CEEISCAT), Badalona, Spain; 2 Institute for Health Science Research Germans Trias i Pujol (IGTP), Badalona, Spain; 3 CIBER Epidemiologia y Salud Pública (CIBERESP), Madrid, Spain; 4 National Institute of Public Health, Trubarjeva 2, Ljubljana, Slovenia; 5 Institute of Public Health of Serbia “Dr Milan Jovanovic Batut”, Belgrade, Serbia; 6 NRC for HIV/AIDS Prevention, Slovak Medical University, Bratislava, Slovakia; 7 Department of Drug and Infectious Diseases Epidemiology, National Institute for Health Development, Tallinn, Estonia; 8 National AIDS Centre, The Agency of the Ministry of Health, Warsaw, Poland; 9 Department of Paediatrics, Obstetrics and Gynecology and Preventive Medicine, Univ Autonoma de Barcelona, Badalona, Spain

**Keywords:** HIV infection, Sexually transmitted infections, Hepatitis, Testing, Linkage-to-care, Community health services, Monitoring and evaluation, Europe

## Abstract

**Background:**

National testing strategy, including monitoring and evaluation, is critical in responding to HIV, sexually transmitted infections, and viral hepatitis. Community-based voluntary counselling and testing contributes to early HIV diagnoses among key populations. Countries providing community-based testing, should integrate some core data on testing and linkage to care in these services into national surveillance and monitoring and evaluation systems. This study aimed to support the integration of community-based voluntary counselling and testing data into respective national surveillance and M&E systems for those infections.

**Methods:**

Preliminary consensus on indicators for the integration of community-based voluntary counselling and testing data into respective national surveillance and monitoring and evaluation systems was reached. Pilot studies were conducted in Estonia, Poland, Serbia, Slovakia, Slovenia and Spain. After pilot activities were implemented, the final consensus on indicators was reached. An analysis of the facilitators and barriers faced during pilot studies was conducted to inform the final recommendations for implementation.

**Results:**

The minimum set of six indicators to be integrated into national surveillance and monitoring and evaluation systems were: number of tests, number of clients tested, reactivity rate for tests and clients, positivity (active infection) rates for tests and clients, linkage to care rates for clients with reactive and/or positive test result, proportion of all new diagnoses in a country with first reactive test result at community-based voluntary counselling and testing service. Seven additional indicators were identified. Each indicator should be disaggregated by key population, sex and age group. A list of 10 recommendations for the collection and integration of community-based voluntary counselling and testing data into national surveillance and monitoring and evaluation systems for HIV, sexually transmitted infections and viral hepatitis was identified.

**Conclusions:**

Integration of some community-based voluntary counselling and testing monitoring and evaluation data into national surveillance and monitoring and evaluation systems in all pilot countries was achieved. The recommendations will support such integration in other European countries. European Centre for Prevention and Control of Diseases included questions from the minimum list of indicators into their Dublin Declaration questionnaire 2020 to contribute to evidence based community testing policies in European countries.

## Background

Timely diagnosis of HIV infection, other sexually transmitted infections (STIs) such as syphilis, gonorrhoea and chlamydia infection, hepatitis B and hepatitis C is a precondition for referral to treatment, which is essential to decrease respective morbidity and mortality and prevent onward transmission of these infections. Evidence-based national testing policies and programmes aiming to reach and test those at risk for these infections have become a public health priority.

Initiatives to improve monitoring of testing and linkage to care have historically focussed on HIV. In October 2016, the European Centre for Disease Prevention and Control (ECDC) convened an expert consultation with representatives from national institutions, community organisations, and healthcare workers from 14 Member States and international organisations, to explore how to strengthen HIV testing and linkage to care monitoring in the European Union/European Economic Area(EU/EEA) countries [1]. The consensus was that improving testing policies, planning, resource allocation and program performance requires timely, accurate and high quality data on HIV testing and linkage to care locally, nationally and within the European region [1]. Recently, the focus has been broadened to include hepatitis B and hepatitis C testing, and ECDC has published Public health guidance on HIV, hepatitis B and C testing in the EU/EEA [2] that recommends testing to be conducted not only in health care settings but also in prisons, drug treatment services, harm reduction services, in the community, and by self-sampling and self-testing. The document also emphasized that an effective national testing strategy, which includes a monitoring and evaluation (M&E) framework, is critical in responding to HIV, hepatitis B and hepatitis C.

Community-based voluntary counselling and testing (CBVCT) has been shown to contribute to a sizeable proportion of new HIV diagnoses, especially among men who have sex with men (MSM) [3–6]. CBVCT services can reach key populations at higher risk of HIV infection and STIs such as MSM, sex workers (SW), people who inject drugs (PWID) and migrants [7]. Guidelines for the monitoring of HIV testing in CBVCT services have been developed by the HIV-COBATEST project [8] and are used throughout Europe, particularly in the COBATEST network, a European network of CBVCT services with the purpose of sharing common data collection instruments to monitor the activity of CBVCTs in Europe, generating, analysing and disseminating harmonised community based testing data and indicators to be used at local, national and regional level [7, 9, 10]. These guidelines promote quality and consistency in data collection, ensuring CBVCT data can be used for M&E within the service and at the national and European level [11, 12]. It is vital that countries collect at least some core data on CBVCT activity to be integrated into national surveillance systems.

The “Joint Action on integrating prevention, testing and linkage to care strategies across HIV, viral hepatitis, tuberculosis and STIs in Europe” (INTEGRATE) aimed to increase early diagnosis and linkage to prevention and care not only for HIV and viral hepatitis, but also for tuberculosis and STIs in EU Member States by 2020. It is a collaborative action among 29 partners from 16 countries participating in the third EU Health programme, funded by the European Union’s 3rd Health Programme for Research and Innovation (Grant Agreement No: 761319). One of the objectives was to support the integration of testing and linkage to care data from CBVCT services into national surveillance and M&E systems for HIV, STIs and viral hepatitis. The work focused on developing a document with consensus recommendations for collection and integration of CBVCT and linkage to care data into national surveillance systems for HIV, viral hepatitis and STIs, including an agreed list of core CBVCT M&E indicators.

This work is turn would support EU/EEA countries to have better informed CBVCT policies and programmes for these infections.

## Methods

Two consensus meetings were held in Barcelona, one before and one after the implementation of pilot activities in six countries (Estonia, Slovakia, Serbia, Poland, Slovenia and Spain). The meetings brought together representatives of public health institutions and CBVCT services from each participating country.

The first consensus meeting was held in Barcelona in December 2018. A preliminary set of CBVCT M&E indicators for HIV, STIs and viral hepatitis was proposed to be integrated into national surveillance and M&E systems. The proposal was based on the outcomes of the Euro HIV EDAT project [8, 11, 12], the Dublin Declaration Monitoring questionnaire 2018 [13] and ECDC Public health guidance on HIV, hepatitis B and C testing in the EU/EEA [14]. The proposal was discussed during the meeting and a preliminary set of CBVCT M&E indicators was agreed upon to be piloted.

The partners (Centre of Epidemiological Studies of HIV/AIDS and STI of Catalonia (CEEISCAT); Institute of Public Health of Serbia “Dr. Milan Jovanović Batut” (IPH), National Aids Centre, Poland (NAC), National Institute of Public Health, Slovenia (NIJZ), Slovak Medical University (SMU), and National Institute for Health Development, Estonia (TAI)) coordinated the implementation of pilot activities in their respective country in the period 1st January 2019 to 31st June 2019. Some pilot activities were extended to 31^st^ August 2019. Pilot activities in each country were planned based on a previous need assessment carried out in each country [15]. Pilot activities varied by country but had the common goal of evaluating the feasibility of implementing the proposed set of indicators.

The second consensus meeting with representatives from CBVCT services, national surveillance and M&E institutions from the six pilot countries was held in Barcelona in November 2019. Each pilot partner (mainly from national surveillance and M&E institutions) invited to the meeting other experts from their institutes or from other surveillance institutes, and a representative from CBVCT services in their countries, involved in the pilot activities. As part of the decision-making process, a proposal of indicators was presented, based on the initial list of indicators proposed to implement during the pilots. The consent decision rule was applied to reach a consensus on the final list of indicators. The proposal was discussed, sharing pilot partner experiences and opinions on the importance and/or feasibility of each indicator. Once the proposal was discussed and a shared understanding was established, the participants were asked to clearly stated if they object or consent to the proposal. In case of any objections, the causes were discussed, and the proposal was amended accordingly, iterating again the previous steps until everyone involved in the decision-making process had no longer meaningful objections.Previous to the meeting, the facilitators and barriers faced by each partner in implementing pilot activities [15] were analyzed, selecting the main ones that were presented in the meeting to open a discussion, in order to inform the recommendations. The same making-decision process and the consent decision rule used for the indicators were applied to reach a consensus on the final recommendations.

For the estimation of indicators in each pilot country de identified data was used.

## Results

The pilot activities in each country were very different between them, and were planned according the needs assessment conducted prior to implementing the pilots. The needs assessment, the detailed planned activities and the results can be consulted in the pilots report document [15]. Table 1 summarize the pilot actions and the main outcomes assessed in each pilot country.

**Table 1:** Summary of the pilot actions and the main outcomes assessed

Countries	Participating CBVCT sites /total number of CBVCT sites	Testing of different diseases	Pilot actions	Main outcomes assessed
Estonia	6/6	HIV	Develop a time efficient way to include HIV rapid testing data from VCTs and CBVCTs/outreach activities into the annual national surveillance report Analyse the 2010–2018 CBVCT HIV rapid testing data and write a report/submit an abstract Making HIV self-testing available at CBVCT sites, and to provide further training for CBVCT personnel on how to counsel clients to use HIV self-testing kits and how to collect data	VCT and CBVCT data added to annual national HIV surveillance report Aggregated data published in the annual national HIV surveillance report Number of HIV tests performed at anonymous HIV testing sites in 2010–2018 CBVCT data during in 2012–2018 analyzed
Poland	30/30	HIV	Linkage of positive test results from community testing database to the national case-based surveillance database using confirmatory test number To include testing for Syphilis and HCV in the electronic data collection system for CBVCT services	Integration of VCT database with HIV case-based surveillance system linking cases by Western Blot number, VCT number and other variables, reducing missing data from HIV surveillance (gender, nationality, age, transmission route) VCT data was integrated into national surveillance report No collection of information on testing for HCV and Syphilis in VCT In August 2019—National Aids Centre’s mandate was extended from HIV/AIDS to STI’s prevention, allowing the collection of STI testing from VCT in the future
Serbia	2/7	HIV	Improvement of voluntary counselling and testing forms to enable identification of community-based testing Use of unique client identifier to monitor linkage to care	Most of the key indicators for HIV were obtained M&E data linked with surveillance data (HIV registry) for those preliminary reactive which are confirmed as HIV positive (and linked to care) Developing a system for automatic personal data coding in HIV registry, in line with Unique Identifier used for online VCT database Matching Unique Identifiers of HIV positive cases in VCT database with HIV register
Slovakia	3/4	HIV, Syphilis, HCV	To implement standardised data collection tools in the CBVCT services, as most of them are not using a questionnaire Discussions and meetings with epidemiologist, stakeholders, National AIDS Committee to discuss about the needs to integrate minimal set of indicators to Epidemiological Information System	Most of key indicators for HIV, Syphilis and HCV were obtained HIV epidemiological reporting system was extended to minimum set of indicators for NGOs Information about HIV-positive persons with first HIV-reactive in NGO was added into Epidemiological Information System Data was included in the yearly National AIDS programme report and also in the National infection disease control Action plans
Slovenia	1/1	HIV, Syphilis, HCV, HVB, Gonorrhoea	Agreement reached between Legebitra (CBVCT service for MSM) and NIJZ: on the list of core testing M&E indicators on the mode for data submission from Legebitra to NIJZ	Testing & linkage to health care data for HIV, STIs, HBV and HCV available at Legebitra has been integrated into the national surveillance and M&E system After 2019, Legebitra will submit the estimates for the agreed core testing M&E indicators to NIJZ annually These results will be included in respective annual reports published by NIJZ
Spain	12/12	HIV, syphilis	Integration of HCV testing for HIV seronegative MSM/trans at high risk into the existing CBVCT Network in Catalonia Implementation of a risk assessment tool HCV testing and risk assessment questionnaire added to data collection tool To promote a national network of CBVCT services in Spain	Most of key indicators for HIV, Syphilis and HCV were obtained At Catalonia level there was involvement of all key stakeholders (CBVCT services, CEEISCAT, ASPCAT) but not at national level. In-person meetings with ministry are still needed

Several facilitators and barriers in the process of integrating CBVCT M&E indicators into the national surveillance and M&E systems for were identified by each pilot country. The summary of the main ones is presented in Table 2.

**Table 2:** Summary of the main barriers and facilitators in the process of integrating CBVCT M&E indicators into the national surveillance and M&E systems identified by pilot countries

Main barriers	Main facilitators
Lack of communication paths Financial and technical capacity of NGOs Anonymity vs. UCI (tracing clients in follow-up) Different UCI and information systems between CBVCT services and the public health systems IT deficits (databases, data collection tools,...) Lack of legal framework for lay providers and data sharing Legal/structural issues (access to healthcare especially for key populations)	Willingness on part of NGOs Suggested indicators already widely used in other settings, so actionable Data already exists in some cases and only processes of data transfer need to be improved Funding and international interest ensured time was freed up to dedicate to these activities Access to free data collection tools Existing relationships between CBVCT and public health (linkage to care) Funding and international interest ensured time was freed up to dedicate to these activities

The 10 consensus recommendations for collection and integration of CBVCT testing and linkage to care data into national surveillance systems for HIV, viral hepatitis and STIs are presented in Table 3.

**Table 3:** Recommendations for collection and integration of CBVCT testing and linkage to care data into national surveillance systems for HIV, STIs and viral hepatitis

1. The country context should be taken into account when interpreting CBVCT M&E data, particularly with regards to the availability of tests for CBVCT services, barriers to testing and barriers to treatment
In order to increase diagnosis and treatment, particularly for key populations, it is crucial that CBVCT services have access to tests and are able to link clients to care following a reactive test result. In some countries there are regulations that prevent PWID diagnosed with hepatitis C from accessing treatment, leading to questions around the ethics of testing people who cannot access treatment when diagnosed with infection. CBVCT services should have access to tests and all clients with a reactive test should be linked to appropriate care and treatment
2. Quality assurance of CBVCT services should be supported
National surveillance and M&E institutions should consider how to support CBVCT services to collect good quality data. Quality assurance in CBVCT services should extend beyond data collection processes and should incorporate promotion of good testing practices, capacity building for staff and volunteers as well as quality assurance of testing kits
3. The contribution of CBVCT services to diagnoses should be recognised
Integrating CBVCT M&E data into respective national surveillance and M&E systems for these infections and disseminating reports will increase recognition of the contribution of CBVCT services in diagnosing these infections. The ECDC’s Dublin Declaration Advisory Group has made steps towards increasing recognition of CBVCT for HIV services at the European level by including questions about community-based testing in their Dublin Declaration Monitoring Questionnaire in 2018 and 2020
4. A unique client identifier can be used by CBVCT services to monitor repeat testers
Use of a unique client identifier is an option for CBVCT services as an alternative to collecting client names. The unique client identifier can be an alphanumeric code based on a number of personal questions which uniquely identify the client while ensuring that the client does not need to remember their code for different visits. Clients who do not wish to provide information for constructing the unique client identifier should be allowed to access testing regardless
5. Using a standardised set of indicators is necessary to ensure data is comparable within a country and between countries
To ensure CBVCT M&E data is comparable among CBVCT services within a country and between countries it is necessary to use a standardized set of indicators. A minimum and extended set of CBVCT M&E indicators has been agreed upon by INTEGRATE project. Pilot activities have demonstrated that the data necessary to obtain estimates for these indicators is feasible to collect in the community setting. When possible, the use of a standardized data collection tool makes it easier to calculate the estimates for the standardised set of CBVCT M&E indicators
6. Before implementing new reporting requirements, national surveillance and M&E institutions should understand what data CBVCT services are already collecting and if it is compatible with the recommended indicators
CBVCT services often already collect data necessary to estimate the recommended set of CBVCT M&E indicators. National surveillance and M&E institutions should try to use available data as far as possible and not unnecessarily increase the data collection and/or reporting requirements. Adding unnecessary reporting requirements uses the CBVCT services’ limited resources and can damage relations between the national surveillance and M&E institutions and CBVCT services
7. The indicator “Proportion of new diagnosis with first reactive test at CBVCT service” is essential to understand the contribution of CBVCT services to diagnosis
It’s important to collect the indicator “Proportion of all new diagnosis in a country with a first reactive test at a CBVCT service” in order to understand the contribution of CBVCT services to diagnosis of these infections. The data for estimating this indicator can be collected in one of the following ways: 1) CBVCT services and surveillance system using a common unique identifier to identify patients linked to care from CBVCT services; 2) CBVCT services collecting self-reported data from clients about confirmatory testing and linkage to care; 3) Estimating the indicator by triangulating the information about the number of reactive tests in CBVCT services and the number of reported new diagnoses to the national surveillance and M&E institution each year. The method of collecting the data to estimate this indicator will depend on the country context
8. All stakeholders must “buy in” to the objective of integrating data
Integrating CBVCT M&E data into the national surveillance and M&E system requires the cooperation of all stakeholders. Clear common goals can facilitate this. For example production of an annual surveillance and M&E report for respective infections that include data about M&E of CBVCT services which is publicly available online and can be utilised by the CBVCT services can contribute to better collaboration
9. The use of online data collection which the national surveillance and M&E system has access to can reduce reporting burden
There are free tools for data collection that can be used by CBVCT services and, with the agreement of CBVCT services, sent to national surveillance and M&E systems. Using an online tool which the national surveillance and M&E system has access to can reduce reporting burden and ensure standardised data. The COBATEST Network has a free data collection tool (access through www.cobatest.org), which is used by the Catalan Network of CBVCT services so that data is harmonised and can be collected and analysed centrally without burdening CBVCT services with extra reporting tasks
10. Where compliance with data protection legislation is an issue, countries can collect estimates for the M&E indicators from CBVCT services instead of collecting disaggregated (case-based) data
In light of the EU General Data Protection Regulation (GDPR), sharing case-based data with the national surveillance and M&E institution may present a problem for CBVCT services if they have not already asked for the client’s consent. Instead of sharing disaggregated (case-base) data with the national surveillance and M&E institution, CBVCT services can calculate and submit estimates of the CBVCT M&E indicators. CBVCT services should be offered technical support when necessary to ensure they can estimate these indicators correctly

The main recommendation is to use for the integration the consensus set of indicators. The recommendations highlight the contribution of CBVCT services to early diagnoses of these infections, and ask for support CBVCT services, in order to get a good quality data. In order to recognize the contribution of CBVCT services to early diagnoses, is important to collect an indicator to know the proportion of new diagnosis with first reactive test at CBVCT service. The cooperation of all parties is essential, and is important to take advantage of the already data collected at CBVCT services, improving the systems, instead of duplicate reporting requirements. One of the problems detected for data integration is the lack of a unique client identifier that could be used through the continuum of HIV prevention and care.

A set of six minimum CBVCT M&E indicators to be integrated into national surveillance and M&E systems and an extended set of seven additional indicators were identified. Respective definitions, numerators, denominators and comments are given in Table 4. All CBVCT M&E indicators in the minimum set are relevant for HIV infection, hepatitis B, hepatitis C and all STIs tested for at CBVCT services.

**Table 4:** Minimum set of CBVCT M&E indicators for HIV, STIs and viral hepatitis to be integrated into national surveillance and M&E systems

1. Number of tests	
Definition	Number of tests performed
Comments	The number of tests corresponds to the number of testing events for a particular infection. Tests used at CBVCT services are usually screening tests. Only rarely are confirmatory tests used. Number of tests is used as a denominator for some of the CBVCT M&E indicators listed below
2. Number of clients tested	
Definition	Number of clients tested
Comments	The number of clients tested corresponds to the number of clients tested for a particular infection. Number of clients tested is used as a denominator for some of the CBVCT M&E indicators listed below
3. Reactivity rate	
a. Reactivity rate of tests	
Definition	Proportion (%) of reactive screening test results among all tests performed
Numerator	Number of screening tests with reactive result
Denominator	Number of screening tests performed
Calculation	(Number of screening tests with reactive result)/(Number of screening tests performed) × 100
b. Reactivity rate for clients	
Definition	Proportion (%) of clients with reactive screening test result among all clients tested
Numerator	Number of clients with reactive screening test result
Denominator	Number of clients tested with a screening tests
Calculation	(Number of clients with a reactive screening test result)/(Number of clients tested with a screening test) × 100
4. Positivity/active infection rate *	
a. Positivity/active infection rate of tests	
Definition	Proportion (%) of confirmed positive test results (confirmed active infections) among all screening tests performed
Numerator	Number of confirmed positive test results (confirmed active infections)
Denominator	Number of screening tests performed
Calculation	[Number of confirmed positive tests results (confirmed active infections)]/(Number of screening tests performed) × 100
b. Positivity/active infection rate for clients	
Definition	Proportion (%) of clients with a confirmed positive test result (confirmed active infection) among all clients tested
Numerator	Number of clients with a confirmed positive test result (confirmed active infection)
Denominator	Number of clients tested with a screening test
Calculation	[Number of clients with a positive confirmed test result (confirmed active infection)]/(Number of clients tested with a screening test) × 100
5. Linkage to care rate	
a. Linkage to care rate for clients with a reactive screening test result	
Definition	Proportion (%) of clients with a reactive screening test result that have been linked to care among all clients tested
Numerator	Number of clients with a reactive screening test result that have been linked to care
Denominator	Number of clients with a reactive screening test result
Calculation	(Number of clients with a reactive screening test result that have been linked to care)/(Number of clients with a reactive screening test result) × 100
Comments	Infection specific definitions for linkage to care from CBVCT service agreed upon in each country should be used. For example, for HIV, linkage to care can be defined as linkage to specialist HIV health care within a month after first reactive screening test for HIV at CBVCT service; or, alternative definition could be linkage to confirmatory testing for HIV within a month after first reactive screening test for HIV at CBVCT service
b. Linkage to care for clients with a positive test result (active infection)	
Definition	Proportion (%) of clients with a positive test result (active infection) that have been linked to care among all clients with a positive test result
Numerator	Number of clients with a positive test result (active infection) that have been linked to care
Denominator	Number of clients with a positive test result
Calculation	(Number of clients with a positive test result (active infection) linked to care)/(Number of clients with a positive test result) × 100
Comments	Positive test result is a positive result of a confirmatory test. Infection specific definitions for linkage to care from CBVCT service agreed upon in each country should be used. For example, for HIV, linkage to care can be defined as linkage to specialist HIV health care within a month after first reactive screening test for HIV at CBVCT service
6. Proportion of all new diagnoses with first reactive test at CBVCT	
Definition	Proportion (%) of individuals with new diagnosis in a country with first reactive (or positive) test result at a CBVCT service
Numerator	Number of clients with a new diagnosis with first reactive (or positive) test result at a CBVCT service
Denominator	Number of all individuals in a country with new diagnosis
Calculation	(Number of clients with new diagnosis with first reactive (or positive) test result at a CBVCT service)/(Number of all individuals in a country with new diagnosis) × 100
Comment	This indicator can be estimated in an ecological way, just knowing the total number of diagnosed people in the country and the total number of people with a reactive test in CBVCT services. Other option to estimate this indicator is collecting in the HIV reporting surveillance system if a first reactive test was obtained in a CBVCT service. In both cases this indicator should be calculated at surveillance level, knowing the number of reactive cases in CBVCT services

*A positive/active infection refers to a confirmed case tested with a confirmatory test. This confirmatory test can be performed in the same CBVCT service, in a laboratory or at the HIV specialist (after linkage to care)

An extended set of indicators recommended for HIV, STIs and viral hepatitis where this testing is offered in CBVCT services was also identified (Table 5). Those indicators in the extended set were considered important for CBVCT services, but no so relevant for the integration in the national surveillance and M&E systems. Some of the indicators in the extended set are only relevant for some of the infections.

**Table 5:** Extended set of CBVCT M&E indicators to be integrated into national surveillance and M&E systems

For HIV, STIs and viral hepatitis	
7. Proportion of clients who reported to have been tested previously	
Definition	Proportion (%) of clients who reported to have been tested previously among all clients tested
Numerator	Number of clients who reported to have been tested previously
Denominator	Number of clients tested
Calculation	(Number of clients who reported to have been tested previously)/(Number of clients tested) × 100
8. Proportion of clients who reported to have been tested during preceding 12 months	
Definition	Proportion (%) of clients who reported to have been tested during preceding 12 months among all clients tested
Numerator	Number of clients who reported to have been tested during preceding 12 months
Denominator	Number of clients tested
Calculation	(Number of clients who reported to have been tested during preceding 12 months)/(Number of clients tested) × 100
9. Proportion of clients with reactive screening test result tested with a confirmatory test	
Definition	Proportion (%) of clients with reactive screening test result who have been tested with confirmatory test among all clients with a reactive screening test result
Numerator	Number of clients with reactive screening test result who have been tested with confirmatory test
Denominator	Number of clients with a reactive screening test result
Calculation	(Number of clients with reactive screening test result who have been tested with confirmatory test)/(Number of clients with a reactive screening test result) × 100
10. Proportion of clients tested at specific venues: office, outreach, self sampling, …	
Definition	Proportion (%) of clients tested at specific venues (office, outreach, self-sampling, …) among all clients tested
Numerator	Number of clients tested at specific venues
Denominator	Number of clients tested
Calculation	(Number of clients tested at specific venues)/(Number of clients tested) × 100
11. Proportion of clients tested for HIV also tested for at least one more infection, either STI or hepatitis B or hepatitis C	
Definition	Proportion (%) of clients tested for HIV also tested for at least one more infection, either STI (syphilis, gonorrhoea or chlamydia) or hepatitis B or hepatitis C, at the same testing visit
Numerator	Number of clients tested for HIV also tested for at least one more infection, either STI or hepatitis B or hepatitis C
Denominator	Number of clients tested for HIV
Calculation	(Number of clients tested for HIV also tested for at least one more infection, either STI or hepatitis B or hepatitis C)/(Number of clients tested for HIV) × 100
Comment	The addition of this indicator was suggested during the review process by the INTEGRATE Steering Committee, in order to reflect the integrated testing across diseases areas
12. Proportion of clients with a reactive HIV test with at least one more reactive result for one more infection, either STI or hepatitis B or hepatitis C	
Definition	Proportion (%) of clients with a reactive HIV test result with at least one more reactive result for one more infection, either STI (syphilis, gonorrhoea or chlamydia) or hepatitis B or hepatitis C at the same testing visit
Numerator	Number of clients with a reactive HIV test result with at least one more reactive result for one more infection, either STI or hepatitis B or hepatitis C
Denominator	Number of clients with reactive HIV test
Calculation	(Number of clients with a reactive HIV test result with at least one more reactive result for one more infection, either STI or hepatitis B or hepatitis C)/(Number of clients tested) × 100
Comment	The addition of this indicator was suggested during the review process by the INTEGRATE Steering Committee, in order to reflect the integrated testing across diseases areas
For STIs and hepatitis C	
13. Proportion of clients who reported to have been previously diagnosed	
Definition	Proportion (%) of clients who reported to have been previously diagnosed among all clients tested
Numerator	Number of clients who reported to have been previously diagnosed
Denominator	Number of clients tested
Calculation	(Number of clients who reported to have been previously diagnosed)/(Number of clients tested) × 100

During the review process of the “Consensus recommendations for collection and integration of CBVCT testing and linkage to care data into national surveillance systems for HIV, viral hepatitis and STIs”[16] document, the INTEGRATE Steering Committee suggested adding two indicators to measure the integration of testing of different diseases. Thus, the following two indicators were also included in the extended set: proportion of clients tested for HIV also tested for at least one more infection, either STI or hepatitis B or hepatitis C; proportion of clients with a reactive HIV test with at least one more reactive result for one more infection, either STI or hepatitis B or hepatitis C).

All indicators should be disaggregated by key population, sex and age group.

## Discussion

In the framework of the INTEGRATE Joint Action, a minimum and extended set of CBVCT M&E indicators for integration into national surveillance and M&E systems for HIV, viral hepatitis and STIs was agreed by consensus. A list of recommendations for CBVCT services and national surveillance and M&E institutions to facilitate such data integration was also agreed.

M&E of CBVCT services is important to evaluate and improve the services and to show its contribution to early diagnosis. The COBATEST network ([7, 10, 17]) has shown the feasibility of collecting harmonised community based testing data and indicators that can be used at local, national and European level. It is vital to take into account the M&E data from CBVCT services and integrate at least some core data into national surveillance systems.

According to the results of the “Monitoring implementation of the Dublin Declaration on partnership to fight HIV/AIDS in Europe and Central Asia in 2018” [18], 41 countries reported community-based HIV testing delivered by medical providers, and 25 reported community-based HIV testing delivered by lay providers. In spite of this, only 23 of the 41 countries reporting community-based HIV testing delivered by medical providers were able to provide the number of tests performed in such settings and just 20 were also able to report positivity rate. None of the 25 countries reporting community-based HIV testing provided by lay-providers were able to provide such CBVCT M&E data.

Two European projects, HIV Community-Based Testing Practices in Europe (HIV-COBATEST) and the European HIV Early Diagnosis and Treatment Project (Euro HIV EDAT) previously developed and recommended a group of core indicators [8] to monitor and evaluate community testing, some of which have been incorporated into previous Dublin Declaration monitoring process rounds and have informed the consensus set of CBVCT M&E indicators presented here. The consensus set of indicators includes the four key metrics for testing monitoring and evaluation recommended by the ECDC Public health guidance on HIV, hepatitis B and C testing in the EU/EEA [14]: number of tests; basic demographic data of person tested (e.g., ages, sex and population group); location/setting of the tests; and number of reactive/positive tests.

The presented minimum and extended set of CBVCT M&E indicators should support national surveillance and M&E systems for HIV, STIs and viral hepatitis in integrating standardized information on testing and linkage to care from CBVCT services in respective countries. Collecting such standardized information will allow the comparison of testing and linkage to care data for HIV, STIs and viral hepatitis between different CBVCT services within and between countries.

This study had a close dialogue with ECDC from the outset, and representatives from the pilot countries also participated in the ECDC Dublin Declaration Advisory Group meeting held in ECDC in December 2019. The proposed set of core M&E indicators on HIV, STI and viral hepatitis testing and linkage to care from CBVCT services to be integrated into national surveillance and M&E systems was discussed along with its inclusion in the Dublin Declaration Monitoring 2020. Questions to obtain estimates for the majority of the minimum set of CBVCT M&E indicators (Number of tests; Number of clients tested; Reactivity rate of tests; Reactivity rate for clients; Linkage to care rate; Proportion of all new diagnoses with first reactive or positive test at a CBVCT service) for HIV have been included in the Dublin Declaration Questionnaire 2020. By collecting these estimates, ECDC and the participating countries will be generating comparable information about the impact of CBVCT services in EU/EEA countries on early diagnosis. This will inform regional policies on HIV testing, diagnosis and linkage to care for key populations (MSM, PWID, sex workers, and migrants) in the region.

The consensus recommendations will guide countries initiating or improving the process of integrating CBVCT testing and linkage to care data for HIV, STIs and viral hepatitis into their surveillance and M&E systems. The target audience for these recommendations is public health professionals who coordinate the development of national guidelines or programmes for HIV, STIs and viral hepatitis, public health professionals responsible for surveillance and M&E systems, CBVCT services, community activists and advocates, commissioners or funders of testing services.

The pilot activities highlighted barriers in the process of integrating CBVCT M&E indicators into the national surveillance and M&E systems. They included lack of communication between the different stakeholders; financial and technical capacity of CBVCT services; information technology (IT) deficits; issues around using a unique personal identifier while maintaining anonymity in CBVCT services and IT or legal difficulties to integrate different databases. Some facilitators which could facilitate the process were also identified: willingness on the part of CBVCT services; existence of the necessary data in some services which only requires improvement to the data transfer processes; existing good relationships between CBVCT services and public health institutions. The consensus recommendations may help to overcome difficulties faced by the countries in the process of integration of CBVCT M&E data into national surveillance and M&E systems.

The pilot activities in all six countries were, by and large, successful in collecting the estimates for CBVCT M&E indicators, particularly for HIV. The short duration of the pilot activities was a barrier in some countries which required major process adaptations. Some of the indicators proved to be difficult or even impossible to be estimated during the pilot period in some countries, due to client anonymity, structural or legal issues. The recommendations take these issues into account and should be considered by countries before starting the process of integrating CBVCT M&E indicators into respective national surveillance and M&E systems. Although the pilots took place in six countries, the heterogeneity of European countries means it is possible that other countries will face barriers for the implementation that have not been identified.

## Conclusions and recommendations

Integration of some CBVCT M&E data into national surveillance and M&E systems in all pilot countries was achieved. The recommendations will support such integration in other European countries.

The authors recommend that all EU/EEA member states with CBVCT services should collect data for at least the minimum set of CBVCT M&E indicators for testing of HIV, STIs and viral hepatitis. If resources permit, and according to national priorities, we recommend that EU/EEA member states also consider collecting annual estimates for the extended set of CBVCT M&E indicators. The authors also recommend the publication of CBVCT M&E indicators in countries’ annual national surveillance and M&E reports, alongside contextual information about CBVCT services.

Integrating CBVCT into the national testing strategy, including the M&E framework, would assist the national response to HIV, STIs and viral hepatitis. Integrating the minimum and extended set of CBVCT M&E indicators into surveillance and M&E systems can help to determine the contribution of CBVCT to diagnosis of HIV, STIs and viral hepatitis in the country and allow the comparison of data between CBVCT services and between countries.

The project will aid the ECDC in improving the data collected in the Dublin Declaration monitoring process regarding CBVCT and will consequently inform evidence-based community testing policies for HIV, STIs and hepatitis B and C in EU/EEA countries.

## Data Availability

Data sharing is not applicable to this article as no datasets were generated or analysed during the current study.

## Abbreviations

CBVCT: Community-based voluntary counselling and testing
ECDC: European Centre for Disease Prevention and Control
EU/EEA: European Union/European Economic Area
IT: Information technology
MSM: Men who have sex with men
M&E: Monitoring and evaluation
PWID: People who inject drugs
STIs: Sexually transmitted infections
SW: Sex workers
WP6: Work package 6
